# Measles outbreak in 2018-2019, Madagascar: epidemiology and public health implications

**DOI:** 10.11604/pamj.2020.35.84.19630

**Published:** 2020-03-19

**Authors:** Marcellin Mengouo Nimpa, Jean Claude Andrianirinarison, Vincent Dossou Sodjinou, Alfred Douba, Yolande Vuo Masembe, Fidiniaina Randriatsarafara, Christiane Bodohanta Ramamonjisoa, Armand Solofoniaina Rafalimanantsoa, Richter Razafindratsimandresy, Charlotte Faty Ndiaye, Julio Rakotonirina

**Affiliations:** 1 World Health Organization Country Office for Madagascar, Antananarivo, Madagascar; 2 Ministry of Health, Ambohidahy, Madagascar; 3 World Health Organization Regional Office for Africa, Brazaville, Congo; 4 Felix Houphouet Boigny University, Abidjan-Cocody, Cote d'Ivoire; 5 Pasteur Institute, Antananarivo 101, Madagascar

**Keywords:** Measles, outbreak, epidemiology, Madagascar

## Abstract

**Introduction:**

In October 4^th^, 2018, a measles outbreak was declared in Madagascar. This study describes the epidemiology of the outbreak and determines public health implications for measles elimination in Madagascar.

**Methods:**

Data have been collected using line list developed for the outbreak. Serum samples were collected within 30 days of rash onset for laboratory testing; confirmation was made by detection of measles immunoglobulin M (IgM) antibody.

**Results:**

A total of 2,930 samples were analysed in the laboratory among which 1,086 (37%) were laboratory confirmed. Measles cases age ranged from a minimum of 1 month to a maximum of 88 years. The median and the mean were 7 years and 9 years respectively. Children between 1 to 9 years accounted for 50.6% of measles cases. Attack rate (39,014 per 1,000,000 inhabitants) and case fatality rate (1.2%) were highest among children aged 9-11 months. A total of 67.2% cases were unvaccinated. As of March 14^th^, 2019, all the 22 regions and 105 (92%) health districts out of 114 were affected by the measles outbreak in Madagascar.

**Conclusion:**

Measles outbreak in Madagascar showed that the country is not on the track to achieve the goal of measles elimination by 2020.

## Introduction

Measles is one of the most contagious diseases of humans [1, 2] with a basic reproductive number (the average number of secondary cases produced by a primary case in a completely susceptible population) of 12-18 [3]. Its causal agent is the measles virus. Measles occurs as a seasonal disease in endemic areas. Transmission is primarily person-to-person by airborne respiratory droplets that disperse within minutes, and transmission can also occur through direct contact with infected secretions [1, 4]. Measles incubation period generally lasts 10-14 days (range, 7-23) from exposure to onset of fist symptoms, which usually consist of fever, malaise, cough, conjunctivitis, and coryza. The characteristic morbilliform rash appears 2-4 days after onset of the early symptom or prodrome. Patients are usually contagious from about 4 days before occurrence of the rash until 4 days after eruption, when the levels of measles virus in the respiratory tract are highest. Prior to the onset of rash, bluish-white Koplik's spots, which are pathognomonic for measles, may be seen in the oral mucosa. In uncomplicated measles cases, patients improve by the third day after rash onset, and have fully recovered 7-10 days after onset of disease. In tropical zones, most cases of measles occur during the dry season, whereas in temperate zones, incidence peaks during late winter and early spring [1]. Before the introduction of measles vaccine in 1963, major epidemics occurred approximately every 2 to 3 years and it is estimated that 30 million cases of measles and more than 2 million deaths occurred globally each year [1]. Measles Rubella elimination initiative launched at international level in 2012 was adopted by Madagascar [5-7]. The last measles outbreak occurred in the country in 2003 [8]. On October 4^th^, 2018, a measles outbreak was confirmed by the National Reference Laboratory. This outbreak started in the capital city, Antananarivo, and extended to all the 22 regions of the Madagascar [9, 10]. This study describes the epidemiology of the outbreak, and determines public health implications for measles elimination in Madagascar.

## Methods

**Data collection:** data have been collected using line list developed for the outbreak. Line list was available in health facilities. Patient's information was recorded on the line list and a blood specimen is collected when he/she visited the health facility. Active search of cases was conducted by community workers for patient who did not visit the health facility. Information of patients was sent to the health facility and patients were asked to visit the health facility for management. Variables in the line list included the name of health district, year, suspected disease, epidemiological week, patient's name, health facility's name, residence place's name, sex, age, date of rash onset, health facility visit date, symptoms (maculopapular eruption, fever, conjunctivitis, cough, coryza), immunization status (vaccinated against measles, date of vaccination, not vaccinated), lab test (blood sample, date of sampling, lab result), outcome (alive, dead, unknown), places visited 2 weeks prior the beginning of the illness, and comment (uncommon sign, hospital, community).

**Laboratory test:** serum samples were collected within 30 days of rash onset for laboratory testing; confirmation was made by detection of measles immunoglobulin M (IgM) antibody at an accredited national measles laboratory using a standard commercial enzyme immunoassay indirect kit [11].

**Case definition:** *a suspected measles case* was defined as (i) any person with generalized maculo-papular rash and fever plus one of the following: cough or coryza (runny nose) or conjunctivitis (red eyes); (ii) any person in whom a clinician suspects measles. *Measles suspected cases at community level* was defined as any person with generalized rash and fever. *Laboratory confirmed measles* was defined as a suspected measles case that is investigated, including the collection of blood specimen, has serological confirmation of recent measles virus infection (measles IgM positive) and had not received measles vaccination in the 30 days preceding the specimen collection. *Measles confirmed by epidemiological linkage* was defined as a suspected measles case that has not had a specimen taken for serologic confirmation and is linked (in place, person and time) to lab confirmed cases; i.e. living in the same or in an adjacent district with a lab confirmed case where there is a likelihood of transmission; onset of rash of the two cases being within 30 days of each other. *A confirmed outbreak of measles* was defined as 3 or more measles IgM positive (laboratory confirmed) cases in a health facility or district in one month [12].

## Results

### Sociodemographic characteristics of measles cases

From September 2018 to March 2019, a total of 112,693 measles cases were registered in Madagascar. The outbreak affected equally males and females. Overall, 56,356 cases (50.0%) were females and 56,061 cases (49.8%) males. The sex was not available for 276 (0.2%) cases. Measles cases age ranged from a minimum of 1 month to a maximum of 88 years. The median and the mean were 7 years and 9 years respectively, and the mode was 1 year. The most affected age group was 1-9 years. Children between 1 to 9 years accounted for 50.6% of measles cases. Also, 78.4% were below 14 years and 89.4% below 20 years old. Case fatality rate and attack rate were high among children less than 5 years (Table 1). Highest attack rates were among children below one year and specifically those between 9-11 months where this was almost 9 times the global attack rate. A total of 75,721 (67.2%) cases were unvaccinated.

**Table 1:** Distribution of measles case fatality and attack rate by age group, Madagascar, September 2018-March 2019

Age groups	Total Population	Number (%) of confirmed cases (lab and Epi link)	Number of Deaths (%)	Vaccinated (%)	Case Fatality Rate (%)	Attack Rate per 1,000,000 inhabitants
< 9 months	789,919	8085 (7.2)	84 (11.2)	1576 (19.5)	1.0	10,234
9-11 months	86,891	3646 (3.2)	43 (5.7)	1091 (29.9)	1.2	39,014
1-4 years	3,862,704	31,049 (27.6)	346 (46.3)	10,261 (33.0)	1.1	8037
5-9 years	3,133,346	25,895 (23.0)	124 (16.6)	8703 (33.6)	0.5	8263
10-14 years	3,975,926	19,556 (17.4)	82 (11.0)	6854 (35.0)	0.4	4918
15-19 years	2,369,757	12,387 (11.0)	27 (3.6)	4632 (37.4)	0.2	5226
≥ 20 years	12,112,093	12,075 (10.7)	42 (5.6)	3855 (31.9)	0.3	997
**Total**	**26,330,637**	**112,693 (100)**	**748 (100)**	**36,972 (32.8)**	**0.7**	**4269**

### Spatiotemporal distribution of measles cases during the outbreak

**Figure 1:**
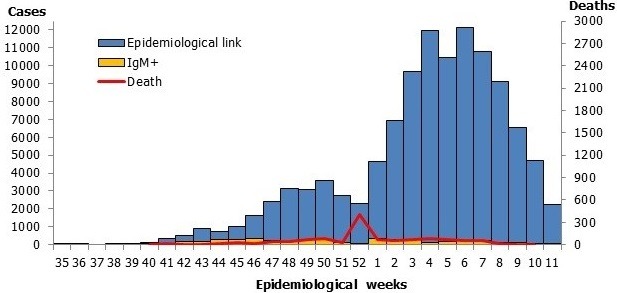
Distribution of confirmed and probable measles cases and deaths by epidemiological week, Madagascar, September 2018- March 2019

**Figure 2:**
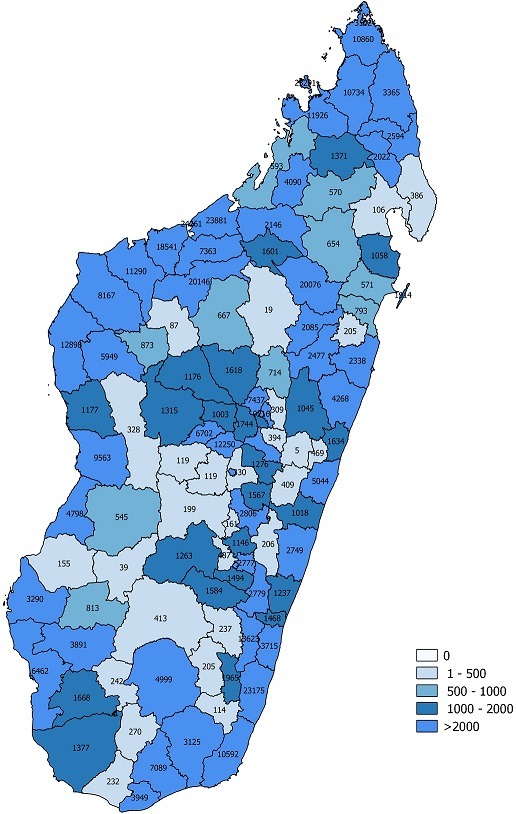
Attack rate per 1,000,000 inhabitants mapping by district, Madagascar, September 2018-March 2019

The outbreak was declared on October 4^th^, 2018. The previous outbreak occurred in Madagascar in 2003. From September 3^rd^, 2018 until March 14^th^, 2019, an estimated 112,693 measles cases were reported including 1086 (1%) laboratory-confirmed cases and 111,607 (99%) epidemiological link-confirmed cases. Over this period, an estimated 748 measles-related deaths (with line list) was reported including 634 health facilities-reported deaths and 114 community-reported deaths. The epidemiological curve is sinuous with two peaks: a small on week 50, and a big on week 6. The death curve is mainly flat with a peak on week 52 of the year 2018 (Figure 1). Spatial distribution shows that the outbreak started in the main district of the capital, Antananarivo, and progressively spread to the nearest districts and finally to all the 22 regions and related districts. As of March 14^th^, 2019, 105 (92%) health districts out of 114 were affected by the measles outbreak in Madagascar. Moreover, the outbreak spread internationally to neighbouring island countries of Comoros and Reunion Island where imported measles cases from Madagascar were also reported. The national attack rate was high with 4269 cases per 1,000,000 inhabitants. Overall, 52 (45.6%) districts had an attack rate greater than 2000 cases per 1,000,000 inhabitants (the vast majority of these districts are seaboard districts which are difficult to access), 24 (21.1%) districts had an attack rate of 1000-2000 cases per 1,000,000 inhabitants, 11 (9.6%) districts had an attack rate of 500-999 cases per 1,000,000 inhabitants, and 27 (23.7) districts had an attack rate of 1-499 cases per 1,000,000 inhabitants (Figure 2).

### Laboratory confirmation

A total of 2,930 samples were analysed in the laboratory among which 1086 (37%) were laboratory confirmed. Five gingival samples were collected during preliminary investigations and were all positive in the national laboratory. Genetic analysis performed by South Africa reference laboratory revealed B3 serotype virus circulation. The virus is 100% closed to the one which has been circulating in France and Europe.

### Clinical manifestations

Most reported symptoms include fever [108,228 (96.04%)], rash [106,164 (94.20%)], cough [95,905 (85.10%)], conjunctivitis [78,266 (69.45%)] and catarrh [73,560 (65.27%)].

## Discussion

Children aged 1-9 years represented half of measles cases during 2018-19 outbreak in Madagascar. While children above one were more represented, measles seems to spread 2 to 9 times more rapidly than the average attack rate in children below one year. The highest attack rate among children between 9-11 months may be due to reduced maternal antibodies acquired at birth in this age group. In Madagascar, cases were mainly children aged less than 5 years. Similarly, most of cases occurred among children under 5 years during a measles outbreak in Nigeria in 2013 [13], Cameroon in 2011 [14], in other African countries other the period 2002-2009 [15], and in Saudi Arabia in 2007. However, in Latin America countries such as Peru and Brazil people aged more than 15 years were most affected [16]. The average age for acquiring measles depends on biological and epidemiological factors, mainly population immunity and birth rate. In certain settings like low income countries or refugee camps, low population immunity, high birth rates and high population density, lead to increased transmission in younger age groups including infants and pre-school children. As vaccination coverage increases, the average age of measles infection can shift to adolescents and young adults [1, 15]. The mean (9 years) and the median age (7 years) were comparable to those reported by Getahun *et al.* in Ethiopia [4] and by Jahan *et al.* in Saudi Arabia [17].

Males and females were equally affected during the 2018-2019 measles outbreak in Madagascar. This trend of equal distribution of measles among males and females was observed in Benin, Niger, Ghana, and Ethiopia [15]. However, in other countries such as Nigeria, Equatorial Guinea, Namibia, Rwanda, Tanzania, and Togo, males were more affected compared to females [13, 15] while in Botswana, Zimbabwe, Mali, Swaziland [15] and Cameroon the majority of cases were females [14]. The variation of sex distribution of cases could be explained by the demographic features in the most affected age groups. In Peru outbreak, Sniadack *et al.* mentioned that being male and aged 16-20 years were risk factors for measles. According to the authors, one explanation for the predominance of young adult males among cases in this community may be that members of this group are more mobile or socially active than their female counterparts or elders [16]. In Madagascar, more than 60% of measles cases were not vaccinated. Measles occurred mainly among unvaccinated children as shown in previous outbreaks [13, 18]. The last measles outbreak registered in Madagascar was in 2003, this is almost 15 years ago. The 2018-2019 outbreak is the most important ever faced by the country. Measles outbreaks occur when the number of susceptible individuals in the population reaches a critical threshold due to low vaccination coverage and high prevalence of malnutrition [4, 19]. The vast majority of measles cases occurred among children aged between 1 to 9 years no longer targeted by the National Immunization Programme. This suggest poor performances of the National Expanded Programme on Immunization (EPI) during the last decade resulted in accumulated number of susceptible children over the years also missed during preventive supplementary immunization activities organized almost every three years.

Global case fatality rate was 0.7 while age specific case fatality rate was greater or equal to 1% among children aged less than 5 years. Most deaths occurred in health facilities at the beginning of the outbreak indicating gaps in the case management despite availability of treatment guidelines. These case fatality rate could be lower if free medicines for case management were available earlier and in adequate quantities in health facilities. However, this case fatality rate was lower than that reported in previous studies. This should be taken cautiously by taking in consideration poor reporting of deaths which occurred in the community due to weak community-based surveillance system. Generally, in Africa, the measles case fatality rate ranges from 3 to 5%, reaching up to 30% during severe outbreaks and outbreaks in closed communities such as refugee camps [4, 20]. The severity of measles varies widely, depending on several host and environmental factors. The risk of developing severe or fatal measles complications increases for children aged <5 years, and persons living in overcrowded conditions, those who are malnourished especially with vitamin A deficiency, and those with immunological disorders, such as AIDS [1, 17]. As of March 14^th^, 2019, 105 (92%) health districts out of 114 were affected by the measles outbreak in Madagascar. The rapid extension of the outbreak to more than 90% of health districts could be explained by 4 factors. Firstly, a large number of susceptible people. Despite measles administrative coverage above 80% at national level since 2014 in Madagascar, the WHO-UNICEF estimates showed a coverage around 58% over the last five years. Secondly, outbreak response immunization (ORI) started 4 months after the official declaration of the outbreak due to resource mobilization issues. In addition, the ORI was implemented by phases due to insufficient measles vaccine stockpiles at global level at the beginning of the outbreak: the first in January, the second in February, and the third in March 2019. Thirdly, the outbreak started in the main city of Antananarivo where there are important populations' movements to other regions. This situation was increased during presidential election. Fourthly, the outbreak started during the presidential election period with high population mobility and numerous mass gatherings activities. This increased close contacts between cases and susceptible people, and could explain both the rapid extension and the high magnitude of the outbreak. In Africa, ORI implementation generally was not rapid and occurred months after the start of outbreaks [21]. More cases can be averted through early intervention targeting a wide age range even if vaccination coverage is low, compared with the number of cases that can be averted through higher coverage with a later intervention [22]. Late intervention highlights the need for Madagascar and other African countries to include in their regular budget, flexible and rapidly mobilizable emergency funds which can help kick off response activities while resource mobilization efforts goes on. Measles cases from Madagascar were exported to a neighbouring country, namely Comoros and Reunion Island and led to local outbreak in these countries. This international spread of the disease reveals the need to strengthen International Health Regulations core capacities in Madagascar within the context of Global Health Security. The country conducted Joint External Evaluation of International Health Regulations (IHR) in 2017, which pointed some gaps that the country started addressing in the surveillance system and entry points. This internal spread also raised the specific challenges in managing outbreak and emergency situation in the cities characterized by population overcrowding, high populations' movements within and outside the country, presence of entry points like airports, and important private sector. Multisectoral and well-coordinated response activities are key for the success in this environment. There is, therefore, an urgent need for better preparedness of main cities to face.

Measles virus isolation and documentation to identify the source is one of the strategies recommended by the World Health Organization Regional Office for Africa (WHO-AFRO) within the context of measles elimination. Madagascar outbreak revealed that B3 serotype circulation in Madagascar is very close to the virus circulating in 2017 in France and Europe which suggest an importation. There are important movements of populations between France and other European countries and Madagascar for economic and touristic reasons. Future research could be conducted on the economic burden of this outbreak. In addition, research should identify the reason of non-vaccination of children in Madagascar.

### Strengths and weaknesses of the study

Strengths of our study stem from method: data have been collected using a standardized tool; cases definition have been standardized; blood samples biological analysis has been performed by the national reference laboratory (The Pasteur Institute of Madagascar) using an internationally accepted technic. The findings of our study are subjected to some limitations. Firstly, we used surveillance database and measles line listing collected from the districts and frontline health facilities. As a result, some key variables were missing for some measles cases like sex or immunization status and there may be underestimation of disease burden in districts poorly reporting their line listing to the central level and those with poor measles surveillance system. Finally, our results may not be representative of all cases of measles knowing that because of financial and cultural barriers, unreported cases and deaths may have been missed in the community.

### Public health implications

This study provides four main lessons with potential implications for public health interest for policy makers, national EPI managers, and partners. Firstly, routine immunization programme has been performing poorly for more than a decade despite very high administrative coverage reported over the years and data quality issues that should be addressed. National forum on routine immunization could be an opportunity for the country and partners to rethink future orientations of the programme. Conducting a national EPI review after that of 2012 and an immunization coverage survey will provide baselines for this routine emergency programme. The place of the private sector in routine immunization implementation should also be discussed as an important part of the population seeks care in private health facilities. Secondly, introducing a second dose of measles vaccines in the national EPI programme is needed to provide a second opportunity to children missed during the first year on age to be vaccinated and reduce susceptible over the years. Thirdly, virus genotype circulating in Madagascar have been identified as B3 virus, which is 100% closed to the virus circulating in France and Europe which may point an importation of the virus. Fourthly, strengthening country IHR core capacities for better preparedness and response capacities for outbreak and emergency situations within the context of global health security remains a challenge for Madagascar, prone to outbreaks and natural disasters.

## Conclusion

Measles outbreak in Madagascar showed that the country is not on the track to achieve the goal of measles elimination by 2020. Much needs to be done to improve immunization performance in order to prevent other large-scale measles epidemics in Madagascar. We recommend a thorough review on the national EPI programme after the last one done in 2012. This will enable in identifying gaps and provide new orientations for better programme management. Secondly, introducing the second dose of measles vaccine in the national programme will provide an additional opportunity for children missed during their first age to get vaccinated and reduce susceptible children. Finally, measles preventive campaign organized every three years as part of measles elimination strategy, should not always be limited to children under 5 years. Identification of preventive campaigns target should take in consideration measles epidemiological profile to determine the most appropriate age group to be targeted in the country in order to prevent large scale outbreaks.

### What is known about this topic

- Measles is one of the most contagious diseases of humans with a basic reproductive number (the average number of secondary cases produced by a primary case in a completely susceptible population) of 12-18;
- The risk of developing fatal or severe measles increases for children aged less than 5 years, living in overcrowded conditions, who are malnourished (especially with vitamin A deficiency);
- Failure to maintain high coverage of childhood immunization in all districts has resulted in a resurgence of the disease, in countries where vaccination has substantially reduced the incidence of measles.

### What this study adds

- Children aged 1-9 years represented half of measles cases during 2018-19 outbreak in Madagascar;
- The early mass immunization campaign against measles could have prevented many cases and the extension of the disease in all the health districts of the country;
- There is a need of having sufficient measles vaccine stockpiles at global level.

## Competing interests

The authors declare no competing interests.
